# Strength Training Among Male Master Cyclists—Practices, Challenges, and Rationales

**DOI:** 10.3390/jfmk9040232

**Published:** 2024-11-12

**Authors:** Vidar Vikestad, Terje Dalen

**Affiliations:** Department of Physical Education and Sport Science, Faculty of Teacher Education and Arts, Nord University, 7600 Levanger, Norway; vidar.vikestad@nord.no

**Keywords:** master cyclists, strength training, endurance performance

## Abstract

**Background:** Cycling performance declines with age due to reduced aerobic capacity, along with reductions in muscle mass and bone density. Strength training can help counter these effects. This study aims to explore the strength training practices, challenges, and decision-making rationale of male master cyclists to optimize performance and health as they age. **Methods:** A total of 555 male master cyclists aged 35 and above completed an online questionnaire, distributed via social media platforms, that included Likert-type, single- and multiple-selection, and open-ended questions. Participants were then divided into two age groups: 35–49 years (n = 359) and ≥50 years (n = 196). Analyses involved descriptive statistics, Wilcoxon signed-rank tests, Mann–Whitney U-tests, and chi-square tests, with qualitative data analyzed using content analysis. **Results:** More cyclists engaged in strength training during the off-/pre-season, with a significant reduction in both frequency and the number of cyclists engaging in strength training during the race season. The strength training practice was focused mainly on core and lower body, employing hypertrophy and maximal strength training methods. Key challenges included fatigue induced by strength training and limited time to perform strength training. The main rationale for the strength training revolved around improving cycling performance, reducing injury risk, and the health benefits of strength training. Both age categories, but the older group in particular, reported bone health as a primary rationale for strength training. **Conclusions:** While strength training offers performance and health benefits, issues of fatigue and time constraints remain substantial, suggesting the need for tailored training programs to improve adherence and effectiveness.

## 1. Introduction

Cycling is a demanding endurance sport that covers disciplines such as road cycling, mountain biking, and gravel cycling. These disciplines are popular among middle-aged athletes, commonly referred to as master athletes. A master’s athlete is typically defined as someone who is over the age of 35 and participates in competitive sports or physical activities [1]. Endurance performance generally declines gradually from age 35, with a more rapid decrease occurring after 50–60 years of age [2]. Aging in endurance cyclists is often associated with reduced maximal oxygen consumption ($\overset{\cdot }{V}O_{2}$ max), peak power output, and maximal heart rate, with the decrease in $\overset{\cdot }{V}O_{2}$ max—primarily due to reductions in maximal heart rate and stroke volume—being a major factor in this decline [2,3,4]. Despite a decrease in aerobic capacity, both first and second ventilatory threshold as a percentage of $\overset{\cdot }{V}O_{2}$ max have been observed to increase with age [3]. In addition to the decline in aerobic capacity, muscle mass also decreases with age (sarcopenia), with the decrease being biggest in fast twitch fibers [5,6]. Despite the effects of aging, it is suggested that exercise can counteract these effects and help preserve physical function and muscle strength [6]. However, the decline in muscle mass and strength commonly associated with aging may partially be due to disuse rather than aging itself, as evidenced by high-level recreational athletes who maintain muscle mass and strength with regular exercise [6,7].

Research suggests that conducting two weekly sessions of heavy strength training can enhance endurance and cycling performance [8,9,10]. These performance gains are often linked to improvements in exercise economy, likely resulting from adaptations in the strength-trained muscles [11]. However, these adaptations seem to diminish if cyclists discontinue strength training for over eight weeks, although they can be sustained with just one session per week [12,13]. These potential benefits from strength training seem to be equal or even bigger for older athletes [14]. In addition to heavy strength training, core stability training is another popular training method, and although it is widely practiced, its effectiveness in enhancing performance remains uncertain. It is hypothesized that it could aid injury prevention [15]; however, some researchers argue that traditional strength exercises may be sufficient to achieve core stability adaptations [16]. A study by Sitko et al. [17] found no improvement in power output for durations ranging from 5 s to 20 min in trained cyclists after a 12-week core training program. Among cyclists, lower back pain and knee pain are the most frequent overuse injuries [18]. Research by Abt et al. [19] indicated that induced core fatigue in competitive cyclists could alter cycling mechanics, potentially increasing the risk of injury by subjecting the knee joint to greater stress.

While several studies have explored the endurance training of professional and elite cyclists, we only found one study that has briefly investigated the strength training practices among cyclists [20,21,22]. In a study involving 97 cyclists, 62% reported regularly engaging in strength training, primarily targeting lower body and core exercises [23]. However, the study did not explore strength training in depth, as it was a secondary focus. A study [24] investigated the strength training practices of long-distance triathletes, reporting that 54.6% of the athletes engaged in strength training. Although comprehensive, this study focused on triathletes rather than cyclists, making its findings less directly applicable to the cycling population. Neither of these studies explored the variations in training practice across different periods of the season.

Although strength training has the potential to enhance endurance performance, incorporating it alongside endurance training can present challenges. Time restrictions and lack of knowledge on how to perform the strength exercises were explained as the primary obstacle for triathletes not engaging in strength training [24]. Similarly, Hoon et al. [23] found that time constraints were the main barrier for cyclists who did not engage in strength training. There can also be some potential challenges when cyclists do perform strength training. If there is not sufficient recovery time between sessions, strength training can have a short-term negative impact on endurance performance [25]. This is believed to result from factors such as neuromuscular fatigue, muscle soreness, and depletion of muscle glycogen [26]. The degree to which endurance performance is affected by strength training may vary depending on the athlete’s prior experience with strength training [27]. To reduce the likelihood of this interference, it is recommended to separate strength and endurance sessions by at least 6 h. However, research has shown that even 24–72 h after the session, strength training can still impair endurance performance [26,28]. Additionally, concurrent endurance and strength training may also interfere with the development of power, strength, and hypertrophy [29].

Despite these potential challenges, strength training is crucial for addressing bone health concerns in master cyclists. Several studies highlight that these athletes tend to have lower bone mineral density than untrained peers of the same age and weight [30,31,32]. Master cyclists not only have lower bone density but also experience a more rapid decline in bone density compared to nonathletes [30]. This issue is further emphasized by Nichols et al. [31], who found that highly trained master cyclists have lower bone mineral density than age-matched nonathletes. Unlike weight-bearing activities such as running, cycling provides minimal benefits to bone health [33,34]. A study by Rector et al. [35] reported that male cyclists had significantly lower bone density and a higher prevalence of osteopenia, with 60% of cyclists affected and a sevenfold greater risk than runners. Strength training, however, has been shown to improve bone density in areas like the lumbar spine and hip in competitive male cyclists [36].

All these factors highlight the potential benefits of strength training for master cyclists to maximize performance and preserve muscle and bone health as they age. Given that age-related declines in aerobic capacity, muscle mass, and bone density are often more pronounced after the age 50, this study compares cyclists aged 35–49 with those over 50 years to capture any differences in strength training practices, challenges, and rationales that may arise as physical changes accelerate [2,5]. Although prior studies have provided insights into strength training practices among endurance athletes, limited research specifically addresses how master cyclists incorporate and sustain strength training, the challenges they encounter, and the rationale behind their choices. This study aims to fill these gaps by exploring three research questions: (1) What are the strength training practices of male master cyclists? (2) What challenges related to strength training do they experience? (3) What is the rationale behind their decision making related to strength training?

## 2. Method

### 2.1. Participants

The sample of male master cyclists included a diverse group of experience levels, ranging from recreational riders to competitive master cyclists; therefore, the sample encompassed diverse experience levels, which allows for a broader understanding of strength training practices. A total of 555 male master cyclists responded to the questionnaire and they were divided into two age categories: 35–49 years for the younger master cyclists (35–49) and ≥50 years for the older master cyclists (≥50). The 35–49 group, consisting of 359 participants, had an average age of 41.8 ± 4.2 years and participated in an average of 8.9 ± 9.7 races annually. The ≥50 group, counting 196 participants, had an average age of 56.9 ± 5.5 years and competed on average 8.7 ± 8.4 races per year. The study was reviewed and approved by Norwegian Agency for Shared Services in Education and Research (SIKT), with reference number 322042.

### 2.2. Questionnaire

Data for this study were collected through a questionnaire designed to explore the training practices, challenges, and rationale for the strength training of male master cyclists. The questionnaire mainly gathered quantitative data, but also included open-ended questions to gather in-depth qualitative responses from participants. The recruitment process involved distributing the questionnaire over the social media platforms Twitter/X, Facebook, Instagram, and YouTube, with the help of individuals already established within the cycling community, such as sport scientists, cycling coaches, cycling influencers, and others who helped share the survey to their cycling audience. The questionnaire received responses from cyclists of all levels, sexes, and age categories. However, the participants of this study were limited to male master cyclists aged 35 and above. Cyclists over 35 years who reported riding for elite or professional teams were therefore excluded.

The questionnaire consisted of 24 questions and was designed by the authors to efficiently gather information on the practices, challenges, and reasoning behind cyclists’ choices regarding strength training. The questionnaire was newly constructed to capture the strength training practices, challenges, and rationales of master cyclists. It underwent pilot testing on a group of male recreational master cyclists, allowing us to refine question clarity and identify any potential biases. The full overview of the questions from the questionnaire is included in the Supplementary Materials for reference.

The start of the questionnaire collected basic demographic information, such as age, sex, and the racing category they participated in (master, elite, professional, etc.). Participants were asked to report the number of race days they had in the previous season and to indicate the duration of effort they felt strongest in, providing insight into their racing demands and strengths. Moreover, the participants were then asked about their weekly strength training frequency for the off-season, pre-season, and race season periods, choosing from nine preset options ranging from 0 to 7 sessions per week. Responses of “less than one session per week” (not zero) were counted as 0.5 sessions for the purpose of calculating average sessions per week in the analysis.

Five of the questions used a 9-point Likert scale, with responses ranging from 1 (“Not content at all”), 5 (“Neutral”), to 9 (“Very content”) for satisfaction-related questions, and from 1 (“I strongly dislike”), 5 (“Neutral”), to 9 (“I very much like”) for enjoyment-related questions. These questions regarded the following topics: contentment with their received coaching for both endurance (1) and strength training (2), enjoyment of endurance (3) and strength (4) training, and a question asking how confident the cyclists were that strength training could improve their cycling performance (5). The cyclists were also asked whether they have experienced improvements in cycling performance as a result of strength training, where they were presented with the alternatives “yes”, “no”, and “not sure”. Additionally, there were six single-selection and multiple-selection questions with preset alternatives. Participants were asked about various aspects, such as strength training methods (1), muscle groups they regularly trained (2), reasons for performing strength training (3), challenges related to strength training (4), and their beliefs regarding the positive (5) and negative effects of strength training (6). Questions were designed to be neutral to minimize bias and enhance the reliability of responses. An example of a neutral question used in the questionnaire is: “What are your primary rationale for performing strength training?”. This has multiple-selection options such as “improving cycling performance”, “reducing injury risk”, and “enhancing overall health”. This question was crafted to allow participants to select options without leading them toward any specific rationale.

### 2.3. Data Analysis

#### 2.3.1. Quantitative Analysis

A quantitative analysis was carried out using IBM SPSS Statistics (Version 28). For the multiple- and single-selection questions, the results were presented as the percentage of the cyclists of each age group selecting each option. Statistical analysis involved applying the Chi-square test, with results displayed in tables that included chi-square (χ^2^) values and corresponding *p*-values, allowing for a thorough examination of associations within various categorical variables. For the scale-based questions, due to the non-normal distribution of the data, the Mann–Whitney U-test was used to compare responses between the two age groups, with z-scores and *p*-values provided. The Wilcoxon signed-rank test was also employed to evaluate whether changes in the number of sessions per week across three periods were statistically significant, presenting z-scores and *p*-values for clarity. Participants who did not engage in strength training were excluded from the average sessions per week calculation. The McNemar test with Bonferroni correction was used to assess differences in categorical data to evaluate within group differences.

#### 2.3.2. Qualitative Analysis

In addition to the quantitative data, the open-ended question where the cyclists could add their additional comments gathered text responses from 120 of the participants, totaling approximately 4000 words. The qualitative data from the questionnaire were analyzed using a deductive content analysis [37], as presented in Table 1, which offers an overview of the key content of the text data from the questionnaire. The analysis began by filtering out responses not relevant to the research questions. Codes were then developed for each research question based on the themes identified from the quantitative questionnaire results. The text data underwent multiple readings by the main author to ensure accurate coding, with the entire content analysis process being guided by advice from an external expert in qualitative research. Table 1 outlines the qualitative data derived from the questionnaire, the associated codes within each of the research questions, and the essence encapsulated by each code. For instance, the code “Execution of Strength Training” delves into the specifics of how strength training routines were performed, encompassing details such as exercise selection and execution. This data provide insights into participants’ strength training practices and shed light on their strength training-related challenges and the rationale behind their training choices.

## 3. Results

### 3.1. Strength Training Practice

**Figure 1:**
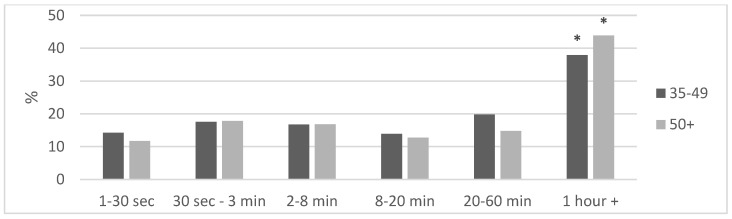
Responses to the question: “What duration of effort do you consider yourself strongest at?”, shown as a percentage of respondents per age group (35–49 and ≥50). Asterisks (*) indicate significant differences from the other alternatives, as determined by the McNemar test, *p* < 0.01.

The Mann–Whitney U-test revealed no significant difference in strength training sessions per week between the two age categories. More cyclists engaged in strength training (≥1 session per week) during the off-season (35–49: 79.1%; ≥50: 82.2%) and pre-season (35–49: 78.9%; ≥50: 82.2%) compared with during the race season (35–49: 59.9%; ≥50: 61.2%). The strength training frequency was lower during pre-season than off-season, and lower during the race season than both off-season and pre-season (Table 2). For both age groups, the most frequent duration of effort the cyclists considered themselves to be the strongest were efforts of one hour or longer, with no difference between the groups, (Figure 1).

### 3.2. Strength Training Method

The lower body and core were the most-trained body parts, while core training, hypertrophy training, and maximal strength training were the most common strength training methods (see Table 3). The ≥50 group reported engaging more in upper body strength training than the 35–49 group. The open-ended questions revealed that the cyclists reported various strength training practices tailored to their specific needs and lifestyles. Many participants emphasized the importance of core strength training and perceived it to play a critical role in improving stability and overall cycling performance. Specific exercises frequently mentioned included deadlifts, squats, leg presses, and various core exercises. Additionally, some cyclists, particularly those who also engaged in other sports like swimming and running, integrated their strength training routines to benefit all their athletic pursuits. Notably, a few cyclists focused on concentric lifts to reduce hypertrophy and maximize muscle recruitment, indicating a strategic approach to their strength training regimen.

### 3.3. Challenges Related to Strength Training

Fatigue/soreness affecting the endurance training and limited time to perform strength training were reported as the main perceived challenges associated with performing strength training (see Table 4). The chi-square test results indicate that there are significant differences in the reported challenges between the two age categories for limited time to perform strength training and other challenges (Table 4). However, no significant differences were found for the remaining factors. Overall, both age categories face similar challenges related to fatigue/soreness affecting endurance training and lack of interest/motivation to perform strength training. However, more of the 35–49 group reported limited time as a constraint, while the ≥50 group more often reported other unspecified challenges.

The text response revealed that the cyclists encountered several challenges in incorporating strength training into their routines. A significant issue was balancing the fatigue induced by strength training with the demands of the endurance training. This attempt to balance strength training with endurance cycling resulted in diverse approaches to exercise routines, repetition ranges, and weight loads. Time management was another critical challenge, with personal and professional commitments often limiting the time available for consistent strength training. Additionally, recovery and injury were recurrent issues, with some participants experiencing difficulties in recovering from strength training sessions, occasionally leading to injuries that impacted their cycling performance. Motivation to engage in strength training also emerged as a common problem, particularly among those who found strength training less enjoyable compared to cycling.

### 3.4. Rationale for Performing Strength Training

The primary rationale for performing strength training among both 35–49 and ≥50 master cyclists was to improve cycling performance, overall health, and reduce injury risk (see Table 5). The ≥50 group reported strength training for improved bone health significantly more than the 35–49 group, while the 35–49 group reported higher perceived benefits of strength training on sprinting performance.

The text responses from the cyclists discovered varied rationales for incorporating strength training into their routines, but it commonly revolved around a few key themes. Injury prevention was a primary motivator, with many participants believing that strength training helped prevent injuries from both cycling and general physical activities. Performance improvement was another significant factor, as cyclists reported that strength training contributed to increased power, endurance, and overall cycling performance, although the extent of improvement varied. For ≥50 cyclists, maintaining muscle mass and counteracting the natural decline in muscle and bone mass associated with aging were critical reasons for engaging in strength training. Lastly, some participants valued strength training as part of a broader fitness regime aimed at maintaining balance, posture, bone health, and overall health.

The two age categories reported no differences in enjoyment of endurance or strength training. Both age groups reported significantly higher enjoyment of endurance training than of strength training. There were also no significant differences between the number of cyclists in each group that received coaching guidance, as 28.1% of the younger and 25.5% of the older cyclists had a coach. However, those who had a coach reported higher enjoyment of both their strength training (Z = −2.28, *p* < 0.05) and endurance training (Z = −4.40, *p* < 0.01), while also being more confident that strength training would improve their cycling performance (Z = −2.11, *p* < 0.05). Among cyclists who engaged in strength training, 69.1% of the 35–49 age group reported noticing improvements in cycling performance from strength training, 2.6% said “no”, and 28.3% were “not sure”. Similarly, in the ≥50 group, 65.3% reported “yes”, 2.9% said “no”, and 31.8% were “not sure”.

## 4. Discussion

The main findings of this study were that the master cyclists typically engaged in two strength training sessions per week during the off-season and pre-season, with a lower frequency of just over one session per week during the race season. A larger proportion of cyclists engaged in regular strength training (≥1 session per week) during the off-season and pre-season compared to the race season. The most reported challenge to perform strength training for both 35–49 and ≥50 master cyclists was managing fatigue, highlighting the difficulty of balancing strength and endurance training required for optimal cycling performance. The most frequently cited rationale for performing strength training was to improve cycling performance, enhance overall health, and reduce injury risk.

### 4.1. Strength Training Practice

Master cyclists commonly performed around two strength training sessions per week during the off- and pre-season, reducing to just over one session per week in the race season. This practice aligns with research recommendations for strength training aimed at improving cycling performance [9,10]. The riders reported that the strength training was focused on lower body and core, using core exercises, hypertrophy training, and maximal strength training. The potential benefits of heavy strength training on cycling performance, as well as on bone health, muscle hypertrophy, and strength, are well established [6,11,36]. The improvement in cycling performance is often explained with improvements in exercise economy, improved rate of force development, reduced or delayed fatigue, and improved lactate threshold and anaerobic capacity [10]. Engagement in core training was also common among the master cyclists; however, studies have not found significant improvements in performance from core training [15,17]. The popularity of core training may stem more from tradition and its simplicity, rather than from evidence of its direct impact on cycling performance. Additionally, core exercises usually do not fatigue the main lower extremity muscles used in cycling, which might make a following cycling session easier than after a heavy leg focused strength training session which can acutely impair cycling performance [26]. It is also suggested that improvement in core strength is only indirectly improving performance in aiding injury resistance [15].

Despite the master cyclists in this study racing significantly less frequently compared to professional cyclists, who often exceed 70 race days annually [38], they still exhibited a marked decrease in strength training frequency from the pre-season to the race season. This observation suggests that, even though the volume of racing for master cyclists is less than professionals, they still reduce their strength training as they approach competition periods. This decrease in strength training frequency during the race season may be due to factors such as prioritizing race-specific cycling training and competitions. Strength training can acutely impair endurance performance, making it less desirable to incorporate strength sessions before high-priority races or key cycling workouts [25]. This could contribute to the challenge of maintaining strength training during the race season. Some research indicates that one weekly maintenance session is adequate to preserve the strength training adaptations; however, most of the adaptations are likely to detrain within the first two months if not maintained [12,13]. Additionally, the warmer and more favorable weather conditions during the race season, which typically occurs in the summer, might contribute to a shift in focus from indoor strength training to outdoor cycling activities, further influencing training decisions. Previous research has demonstrated that cycling tends to be a highly weather-dependent activity [39]. It remains unclear whether this reduction is primarily due to a strategic shift in training focus, time constraints, other factors such as increased fatigue or scheduling conflicts, or simply a preference for outdoor riding during more enjoyable climatic conditions.

### 4.2. Challenges to Performing Strength Training

The challenge of managing fatigue from strength training was the most common issue reported by both the 35–49 and the ≥50 master cyclists. This challenge is significant because it underscores the difficulty of balancing strength training with the endurance training required to perform well on the bike. In this study, fatigue and soreness from strength training were identified as major challenges for the participating cyclists, impacting their ability to perform effectively in endurance training sessions. Muscle soreness from strength training is found to impair endurance performance for up to 72 h [26,28]. This poses a challenge when trying to incorporate strength training, while also optimizing for performance on the cycling sessions.

Time constraints emerged as the second most frequently reported challenge for performing strength training among master cyclists, with this issue being significantly more prevalent in the 35–49 group compared to the ≥50 group. This likely reflects differences in life circumstances, as master cyclists aged 35–49 are often in the midst of career development and may have young families, both of which limit their time for additional training activities like strength training. In contrast, cyclists aged 50 and above may have fewer professional and familial obligations, allowing for more flexibility. This challenge is consistent with findings from non-strength-training triathletes and cyclists, who also cited time limitations as a major barrier [23,24].

Lack of interest or motivation was also a significant challenge for many master cyclists regarding strength training routines, highlighting the motivational barriers to integrating strength training into their routine. This might reflect the finding that the cyclists reported significantly higher enjoyment of the endurance training than strength training. However, the study also found that master cyclists who worked with a coach reported greater enjoyment of both their strength and endurance training. It has previously been suggested that coaching can have a positive influence on intrinsic and self-determined extrinsic motivation [40]. Although these findings emphasize the importance of coaching, it is also possible that athletes who are more motivated and enjoy exercising more could be naturally more likely to seek coaching guidance.

### 4.3. Rationale for Strength Training Choices

Most of the riders in this study performed strength training with the aim of improving cycling performance, enhancing overall health, and reducing injury risk. These findings suggest that master cyclists are motivated by both the performance-enhancing effects and the broad health benefits of strength training. As age progresses, master cyclists face declines in aerobic capacity and muscle mass, particularly in fast-twitch fibers [5,6]. However, strength training has been shown to be effective in counteracting these declines by preserving muscle mass and strength [6,7]. Additionally, strength training has been shown to improve endurance performance. Regular heavy strength training sessions can lead to enhanced neuromuscular efficiency, delayed activation of less efficient muscle fibers, and improved muscle stiffness, all contributing to better cycling performance [8,10]. These physiological adaptations, along with the potential to reduce injury risk, highlight the multifaceted benefits of strength training for master cyclists, particularly as they age.

A significant proportion of master cyclists, especially those in the older age group, reported that the aim of improving bone health was a rationale for their engagement in strength training. This aligns with the existing literature, highlighting bone health concerns among cyclists [30,31,32]. These studies show that master cyclists have lower bone mineral density than their untrained peers, even when controlling for age and weight. This issue is compounded by the fact that cyclists tend to experience a more pronounced decline in bone mineral density with aging compared to nonathletes [30]. Cycling, as a non-weight-bearing activity, does not provide sufficient mechanical loading to stimulate bone formation, unlike weight-bearing exercises such as running or strength training [33,34]. This might explain why older master cyclists may prioritize strength training to counteract the low bone mineral density associated with cycling and to mitigate the increased risk of osteopenia and osteoporosis. Indeed, strength training has been shown to improve bone mineral density in competitive male cyclists, particularly in the lumbar spine and hip regions [36]. Thus, the observed emphasis on strength training for bone health among older cyclists reflects an informed response to these documented risks. These findings suggest that master cyclists are motivated by both the performance benefits and the broader health advantages of strength training.

### 4.4. Limitations

This study relies on self-reported data, which could affect accuracy since it relies on the participant’s ability to accurately recall and report information. However, these data are easy to distribute and can reach a large number of participants quickly. The study might also experience timing and recency bias, since data were gathered during a period which for many of cyclists is considered the off-/pre-season (November/December), which is a part of the period where strength training often is prioritized more than, for example, during the race season. Nevertheless, these investigations may provide direct insight into the participants’ personal experiences, which might not be easily observable. Together with the fact that the questionnaire can be completed anonymously, it may encourage more honest and open responses. On the other hand, this study could also have benefited from a more detailed collection of the cyclist’s endurance training data, in addition to the strength training data.

### 4.5. Practical Application

Master cyclists have strong incentives to maintain consistent strength training due to its well-documented benefits for cycling performance, bone health, and preserving muscle mass as they age. However, the most common challenges identified in this study were managing fatigue from strength training and dealing with time constraints. To address these challenges, cyclists and coaches should focus on strength training programs that minimize fatigue and fit within the athletes’ busy schedules, especially during the race season. Given that the primary rationales for strength training among master cyclists are to improve performance, enhance overall health, and reduce injury risk, by adopting tailored, time-efficient routines, cyclists can optimize their performance while attaining key health benefits like bone density and muscle mass.

## 5. Conclusions

The master cyclists reported a higher strength training frequency during the off-/pre-season than during the race season. More cyclists engaged in strength training during the off-season and pre-season than during the race season. The cyclists mainly targeted the lower body and the core with their strength training. Moreover, the most frequently reported strength training methods were core, hypertrophy, and maximal strength training. Fatigue from strength training, limited time, and lack of motivation to perform strength training was reported as the biggest challenges, with the 35–49 group reporting time restriction to a larger degree than the ≥50 group. Their rationale for engaging in strength training revolved around improving cycling performance, overall health, and reducing injury risk. Significantly more cyclists aged 50 and above reported improving bone health as a rationale for their engagement in strength training compared to the 35–49 age group.

## Figures and Tables

**Table 1:** Overview of content analysis.

Research Question	Codes	Essence of Codes	Number of Responses
Strength training practice	Maximal strength training	Engage in maximal strength training.	5
	Hypertrophy/muscle growth	Focus on hypertrophy to increase muscle size.	3
	Core training	Focus on core training for stability and cycling performance.	8
	Execution of strength training session/exercises	Details on how exercises and strength training sessions are performed.	7
Challenges related to strength training	Fatigue	Fatigue from strength training negatively affect cycling training and performance.	6
	Time restrictions	Time constraints hinder consistent strength training.	9
	Enjoyment/motivation	Struggle with motivation for strength training.	4
Rationale for Strength Training Choices	Cycling performance	Engage in strength training to improve cycling performance.	10
	To reduce injury risk	Perform strength training to lower injury risk.	7
	For overall health	Engage in strength training to improve overall health.	5
	Bone health	Focus on strength training for bone health.	2
	To increase muscle mass	Increasing muscle mass is a goal for some cyclists.	3

**Table 2:** Wilcoxon single rank test comparing the differences in sessions per week between off-season, pre-season, and race season for the two age categories (35–49 vs. ≥50).

Sessions/Week	Off-Season %		Pre-Season %		Race Season %	
	35–49	≥50	35–49	≥50	35–49	≥50
0	18.1	14.2	18.3	15.8	30.6	23.5
<1	2.8	3.6	2.8	2	9.5	15.3
1	9.7	8.7	15.3	12.8	25.1	21.9
2	38.4	36.2	39	37.8	21.2	27.6
3	21.4	23	17.5	17.9	10	6.6
≥4	9.6	14.3	7	13.8	3.6	5.1
Mean sessions/week	2 *^§^	2.1 *^§^	1.8 *	2 *	1.2	1.3

Note: * = higher than race-season (*p* < 0.01), ^§^ = higher than pre-season (*p* < 0.05). Cyclists reporting zero sessions per week were excluded from mean sessions/week values. Cyclists that reported <1 counted as 0.5 sessions per week.

**Table 3:** Responses to the following questions for the two age categories (35–49 vs. ≥50): “What body parts do you train when strength training?”; “What type of strength training do you frequently perform?”. n = total number of respondents, % = percentage of respondents selecting each alternative, x^2^ = chi-squared, n/s = not significant, * = statistically significant difference between age groups at *p* < 0.05 level.

	35–49 n = 359	≥50 n = 196	Total n = 555	Age Differences	
	%	%	%	x^2^	*p*-Value
What body parts do you train during training?					
Upper body	49.9	58.7	53.0	3.95	<0.05 *
Core	68.5	67.9	68.3	0.03	n/s
Lower body	73.8	74.0	73.9	<0.01	n/s
What type of strength training do you frequently perform?					
Maximal strength training (<6 repetitions)	45.7	39.3	43.4	2.11	n/s
Hypertrophy training (6–30 repetitions)	49.6	53.6	51.0	0.81	n/s
Explosive strength training (Lower resistance with maximum movement speed)	21.4	17.9	20.2	1.01	n/s
Core and stability training	60.2	60.7	60.4	0.02	n/s
Blood flow restriction training	1.4	0.0	1.3	2.75	n/s
Cross fit training	9.2	6.6	8.3	1.09	n/s
Other forms of strength training	3.6	4.1	3.8	0.07	n/s

**Table 4:** Challenges related to strength training and perceived negative effects of strength training for the two age categories (35–49 vs. ≥50). n = total number of respondents, % = percentage of respondents selecting each alternative, x^2^ = chi-squared, n/s = not significant, * = statistically significant difference between age groups at *p* < 0.01 level.

	35–49 n = 359	≥50 n = 196	Total n = 555	Age Differences	
	%	%	%	x^2^	*p*-Value
Challenges to performing strength training					
Fatigue/soreness affecting the endurance training	53.2	45.9	50.6	2.69	n/s
Limited time to perform strength training	40.4	28.6	36.2	7.67	<0.01 *
Traveling to races and training camps	4.2	4.1	4.1	<0.01	n/s
Restarting strength training after stage races/racing periods	9.5	8.7	9.2	0.10	n/s
Lack of knowledge on how to perform strength training	10.9	11.2	11.0	0.02	n/s
Coach lacking knowledge on how to perform strength training	0.8	1.0	0.9	0.05	n/s
Lack of interest/motivation to perform strength training	20.1	24.5	21.6	1.47	n/s
Lack of training equipment/facilities	12.0	7.1	10.3	3.22	n/s
Other challenges	3.1	10.2	5.6	12.26	<0.01 *
Negative effects of strength training					
Impaired cycling performance	5.3	6.1	5.6	0.17	n/s
Soreness impairing endurance training	66.6	60.2	64.3	2.24	n/s
Increased risk of injury	16.2	12.8	15.0	1.15	n/s
Increased muscle mass	23.1	19.4	21.8	1.04	n/s
No negative effects	22.3	29.1	24.7	3.15	n/s

**Table 5:** Rationale for performing strength training and believed positive effects of strength training for the two age categories (35–49 vs. ≥50). n = total number of respondents, % = percentage of respondents selecting each alternative, x^2^ = chi-squared, n/s = not significant, * = statistically significant difference between age groups at *p* < 0.01 level.

	35–49 n = 359	≥50 n = 196	Total n = 555	Age Differences	
	%	%	%	x^2^	*p*-Value
Rationale for performing strength training					
To improve cycling performance	72.4	67.9	70.8	1.28	n/s
To reduce injury risk	59.6	58.2	59.1	0.11	n/s
For overall health	67.1	70.9	68.5	0.84	n/s
To increase muscle mass	29.5	27.0	28.6	0.38	n/s
Coach/team recommendation	7.8	3.6	6.3	3.84	n/s
Rehabilitation from injury	12.3	14.8	13.2	0.72	n/s
To improve bone health	33.4	45.4	37.7	7.75	<0.01 *
Other reasons	4.2	3.6	4.0	0.12	n/s
Believed positive effects of strength training					
Improved cycling performance	85.5	81.1	84.0	1.82	n/s
Improved sprinting performance	51.3	38.8	46.8	7.93	<0.01 *
Reduced risk of injury	69.9	68.4	69.4	0.14	n/s
Increased muscle mass	54.9	46.4	51.9	3.62	n/s
Improved bone health	51.8	60.2	54.8	3.61	n/s
Improved overall health	79.7	79.6	79.6	0.00	n/s
No positive effects	0.3	0.5	0.4	0.19	n/s

## Data Availability

The data supporting the reported results are available from the main author upon reasonable request.

## Supplementary Materials

The following supporting information can be downloaded at: https://www.mdpi.com/article/10.3390/jfmk9040232/s1.
