# Supplementary material for: Strength Training Among Male Master Cyclists—Practices, Challenges, and Rationales
Source: J Funct Morphol Kinesiol. 2024 Nov 12;9(4):232. doi: 10.3390/jfmk9040232 (PMC11586982; doi:10.3390/jfmk9040232)
Supplement: Supplementary file 1 [file jfmk-09-00232-s001.zip › jfmk-3248103-supplementary.pdf]

Q.nr.	Question	Question type	Alternatives
1	Year of birth	Type number	Year of birth
2	Sex	Single select	Male/female
3	What is your current level of competition and team affiliation?	Single select	Junior, Club/elite team, Masters/>35, CTM - UCI CONTINENTAL TEAMS, PRT - UCI PROTEAMS, WTT - UCI WORLDTEAMS, CTW - UCI WOMEN'S CONTINENTAL, WTW - UCI WOMEN’S WORLDTEAMS
4	How many days do you reckon you competed/raced the previous season?	Type number	Type number
5	At what duration of effort would you consider yourself the strongest?	Multiple select	1-30 sec, 30sec-3min, 3-8min, 8-20min, 20-60min, 1h+
6	Do you have a coach/coaches planning your training or giving you guidance on your training?	Single select	Yes/No
7	Do you have a separate coach planning or giving you guidance on your strength training?	Single select	Yes/No
8	Does your coach incorporate strength training into your training plan?	Single select	Yes/No
9	How content are you with the guidance/help you receive from your coach/coaches for your strength training?	Likert scale	1-9 (1= Not content at all, 5=Neutral, 9= Very content)
10	How content are you with the guidance/help you receive from your coach/coaches for your endurance training?	Likert scale	1-9 (1= Not content at all, 5=Neutral, 9= Very content)
11	How much would you say you enjoy/like to perform strength training?	Likert scale	1-9 (1=I strongly dislike strength training, 5=Neutral, 9=I very much like strength training)
12	How much would you say you enjoy/like to perform cycling training?	Likert scale	1-9 (1=I strongly dislike endurance training, 5=Neutral, 9=I very much like endurance training)
13	Strength training frequency off-season	Single select	Sessions/week: 0-7 (0, <1, 1, 2, 3, 4, 5, 6, 7)
14	Strength training frequency pre-season	Single select	Sessions/week: 0-7 (0, <1, 1, 2, 3, 4, 5, 6, 7)
15	Strength training frequency race-season	Single select	Sessions/week: 0-7 (0, <1, 1, 2, 3, 4, 5, 6, 7)
16	Which muscle groups do you primarily	Multiple select	Upper body-Core-Lower body

	target in your strength training routine?		
17	What types of strength training do you frequently perform?	Multiple select	Maximal strength training <6 repetitions - Hypertrophy training 6-30 repetitions - Explosive strength training (fast movement with lighter weight) - Core and stability training - Blood Flow Restriction/Occlusion Training - CrossFit Training - Other
18	Rationale for performing strength training	Multiple select	To improve cycling performance - To reduce the risk of injuries - To improve overall health/fitness - To increase muscle mass - Because of coach or team recommendation - Rehabilitation from injury - To improve bone health - Other
19	What challenges do you /would you encounter in maintaining a consistent strength training routine alongside your cycling training and racing?	Multiple select	Fatigue/soreness affecting the endurance training - Can't find the time to do strength training - Traveling to races and training camps - Restarting strength training after stage races/racing period - Lack of knowledge on how to perform strength training - Coach lacking knowledge on how to perform strength training - Lack of interest/motivation to do strength training - Lack of training equipment/facilities - Other
20	What POSITIVE effects do you believe that the inclusion of strength training into your program could provide you?	Multiple select	Improved cycling performance - Improved sprinting performance - Reduced the risk of injuries - Increased muscle mass - Improved bone health - Improved overall health - None
21	What NEGATIVE effects do you believe that the inclusion of strength training into your program could provide?	Multiple select	Impaired cycling performance - Soreness/fatigue negatively effecting endurance training/development - Increased risk of injuries from strength training - Increased muscle mass/body weight - None
22	How confident are you that strength training can improve cycling performance?	Likert scale	1-9 (1=Not at all confident, 5=Neutral, 9=Very confident)
23	Have you noticed any improvements in your cycling performance as a result of incorporating strength training?	Single select	Yes/No/Not sure
24	Please provide any additional comments or insights about your experiences with strength training and its impact on your cycling performance	Type text	Text response
